# Solitary fibrous tumor of the chest wall with Doege–Potter syndrome: a case report

**DOI:** 10.3389/fonc.2025.1630793

**Published:** 2026-01-07

**Authors:** Hongfei Zhang, Xinyue Weng, Yuqi Zhang, Yuchen Wang, Jiangyue Liu, Yutao Pang, Ang Li, Boyun Deng, Fasheng Li, Jie Chen, Zhu Liang, Zhan He, Dong Wu, Zhuming Chen

**Affiliations:** 1 Department of Thoracic Surgery, Affiliated Hospital of Guangdong Medical University, Zhanjian, China; 2 Guangdong-Hong Kong-Macau Institute of Central Nervous System (CNS) Regeneration (GHMICR), Jinan University, Guangzhou, China; 3 Orthopaedic Center, The second Affiliated Hospital of Guangdong Medical University, Guangdong Medical University, Zhanjiang, China

**Keywords:** solitary fibrous tumor, Doege–Potter syndrome, chest wall, hypoglycemia, vascular embolization, thoracic surgery

## Abstract

**Objective:**

To describe the management of a rare chest wall solitary fibrous tumor (SFT) complicated by Doege–Potter syndrome (DPS), emphasizing the critical roles of multidisciplinary consultation and preoperative vascular embolization in optimizing outcomes.

**Methods:**

A 56-year-old male presented with exertional dyspnea, dizziness, recurrent hypoglycemia, and hypokalemia. Contrast-enhanced computed tomography (CT) identified a large chest wall mass (19.2 × 13.3 × 21.6 cm), with biopsy confirming SFT. Multidisciplinary evaluation established a diagnosis of DPS. The patient underwent three cycles of neoadjuvant chemo-immunotherapy (albumin-bound paclitaxel, cisplatin, and anlotinib), followed by preoperative tumor vascular embolization. Surgical intervention involved en bloc tumor resection, left upper lobe wedge resection, and partial resection of the fifth rib.

**Results:**

Neoadjuvant therapy resulted in stable disease per RECIST 1.1 criteria. Preoperative embolization significantly reduced intraoperative blood loss to 200 mL, enabling an uneventful surgery. Postoperatively, hypoglycemia and hypokalemia resolved, and lung re-expansion was satisfactory. As of December 31, 2024, outpatient follow-up revealed no evidence of recurrence or metabolic abnormalities.

**Conclusions:**

This case highlights the importance of recognizing DPS as a rare manifestation of chest wall SFT and underscores the value of multidisciplinary strategies in managing large tumors. Preoperative vascular embolization effectively minimized surgical risks and corrected metabolic disturbances, facilitating successful resection. Further studies are warranted to refine therapeutic approaches for this uncommon clinical entity.

## Introduction

Pleural solitary fibrous tumor (pSFT) is a rare neoplasm that may originate from the visceral or parietal pleura, accounting for approximately 4% of thoracic tumors (1). Most pSFTs are clinically benign; however, in rare instances, pSFT can be associated with Doege–Potter syndrome (DPS), characterized by severe, refractory hypoglycemia (2–4). Current evidence suggests that the hypoglycemia in DPS is primarily driven by the excessive secretion of insulin-like growth factor-II (IGF-II) by pSFT (5–8). Reports of pSFT associated with DPS are scarce, and surgical resection of the tumor is widely recognized as the most effective treatment. This case report details the diagnosis and management of a patient with a chest wall pSFT complicated by DPS. By reviewing and analyzing the existing literature on pSFT with DPS, we discuss the benefits of preoperative multidisciplinary consultation and interventional embolization for large chest wall solitary fibrous tumors, aiming to provide insights for future clinical research and practice.

## Case report

**Figure 1:**
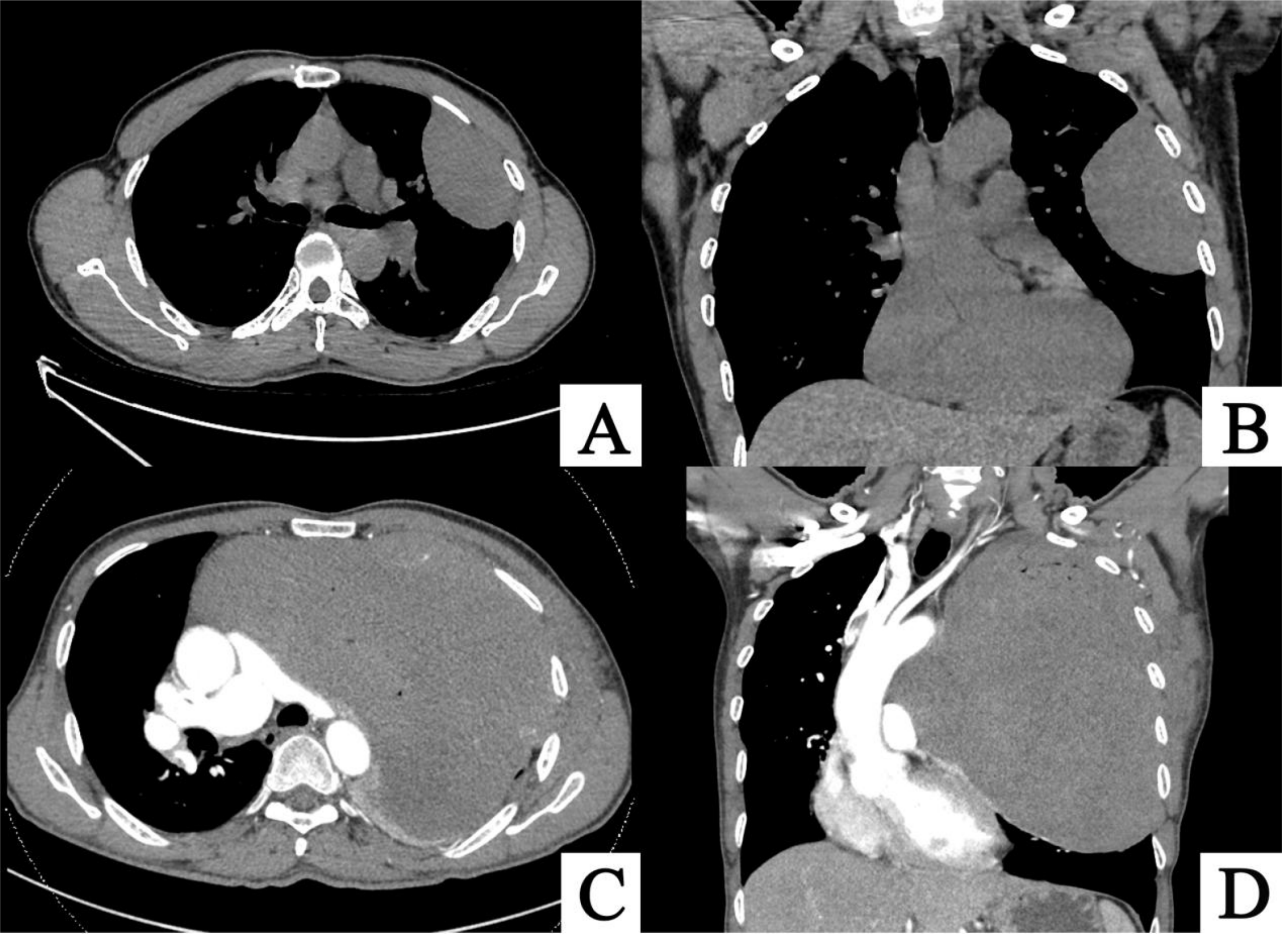
**(A, B)** CT images from August 19, 2019, showing a spindle-shaped soft tissue density mass in the left thoracic cavity, measuring approximately 7.6 cm × 4.6 cm × 9.1 cm, with compression of the surrounding lung tissue. **(C, D)** CT images from May 29, 2024, demonstrating a significant increase in the size of the spindle-shaped soft tissue mass in the left thoracic cavity, now measuring approximately 19.2 cm × 13.3 cm × 21.6 cm. The lesion extends toward the mediastinum, causing a rightward shift of mediastinal structures. The main pulmonary artery and right pulmonary artery are compressed and flattened, with notable progression of left lung atelectasis compared to the prior scan.

**Figure 2:**
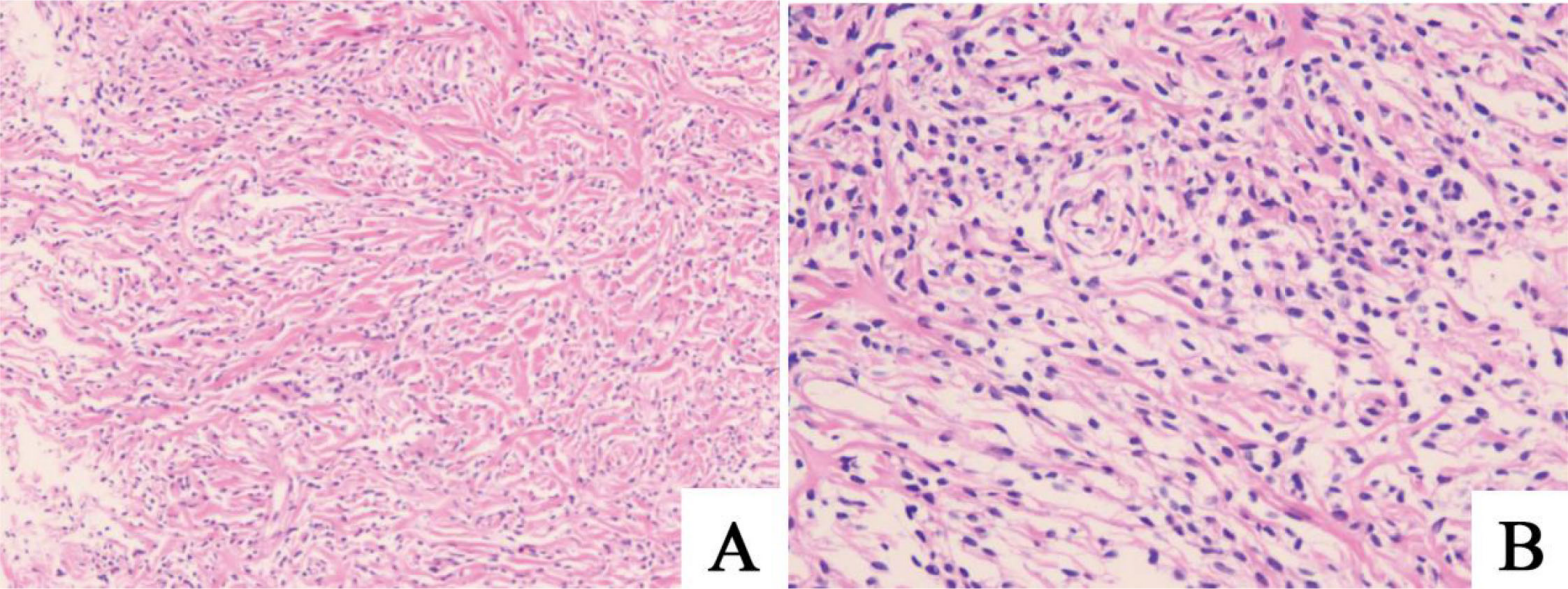
**(A, B)** Histological examination of the chest wall mass biopsy reveals a tumor composed of spindle-shaped cells with dense collagen fibers. Immunohistochemical findings are consistent with a solitary fibrous tumor. Immunohistochemistry results: CK (-), Vimentin (+), STAT6 (+), CD34 (focally +), SMA (-), Desmin (-), S-100 (-), Beta-catenin (cytoplasmic +), LEF-1 (partially +), Ki67 (approximately 5% in hotspot areas).

A 56-year-old male patient presented to our outpatient clinic on August 19, 2019, with a two-week history of exertional dyspnea and dizziness. Chest computed tomography (CT) revealed a soft tissue mass in the left chest wall, and surgical treatment was recommended. However, the patient declined surgery due to personal reasons. On May 29, 2024, the patient returned to our clinic with worsening symptoms. A repeat chest CT showed a significant increase in the size of the left thoracic soft tissue mass compared to the previous scan (**Figure 1**). Upon admission, further investigations were conducted. Laboratory results revealed a potassium level of 3.00 mmol/L(3.5~5.5mmol/L), cytokeratin 19 fragment of 5.160 ng/mL(0~3.3ng/mL), and squamous cell carcinoma antigen of 3.17 ng/mL(0~2.7ng/mL). Routine blood tests, liver and kidney function, coagulation profile, and brain magnetic resonance imaging (MRI) showed no significant abnormalities. To establish a definitive diagnosis, an ultrasound-guided mediastinal mass biopsy was performed. Pathological examination, combined with immunohistochemistry, confirmed the diagnosis of solitary fibrous tumor (**Figure 2**).

Following a multidisciplinary discussion, we concluded that the large tumor volume posed a high surgical risk, making direct resection inadvisable. The patient was subsequently referred to the Department of Pulmonary Oncology for chemotherapy combined with anti-angiogenic targeted therapy. On June 11, 2024, the patient began neoadjuvant chemo-immunotherapy with a regimen of albumin-bound paclitaxel (400 mg), cisplatin (60 mg) on days 1–2, and anlotinib (12 mg) for three cycles, supplemented with hepatoprotective, gastroprotective, and antiemetic supportive care. The third cycle was completed on July 31, 2024.

**Figure 3:**
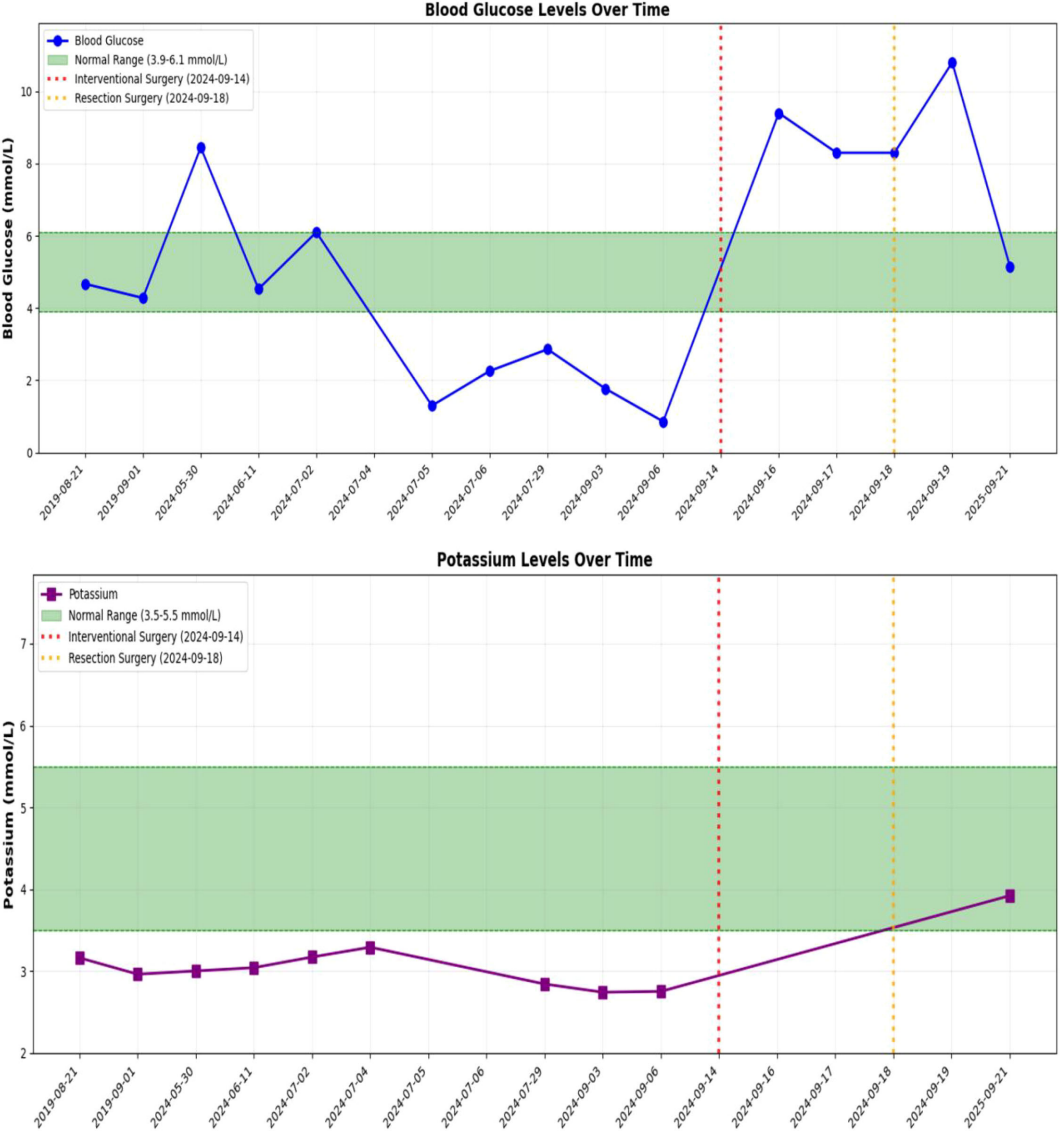
Changes in serum potassium ion concentration and blood glucose concentration before and after surgery in patients.

During treatment, the patient experienced recurrent hypokalemia and hypoglycemia. The lowest recorded serum potassium level was 2.74 mmol/L(4.4mmol/L~6.1mmol/L), and the lowest fasting fingertip blood glucose level was 0.86 mmol/L (**Figure 3**). These symptoms were managed with symptomatic treatment, leading to resolution, and the patient reported no discomfort. One episode of altered consciousness occurred during the second treatment cycle, which resolved spontaneously (A capillary blood glucose of 1.3 mmol/L was documented during the episode.). The patient’s family reported multiple prior episodes of altered consciousness. We hypothesized that these episodes were related to hypoglycemia; however, the patient declined further diagnostic evaluation. No other severe adverse reactions were observed. Follow-up CT imaging showed no significant change in tumor size, with a therapeutic response evaluated as stable disease (SD) per RECIST 1.1 criteria.(RECIST 1.1 evaluation: the single target lesion had a baseline longest diameter of 194.8 mm; after three cycles of neoadjuvant therapy it measured 198.2 mm, representing a +1.7% change).

To facilitate surgical intervention, the patient was transferred to our department on September 2, 2024. Comprehensive endocrine evaluations were conducted to investigate the etiology of recurrent hypoglycemia and hypokalemia, including measurements of cortisol, urinary 17-ketosteroids, aldosterone, adrenocorticotropic hormone, 24-hour urinary potassium excretion, and growth hormone. No significant abnormalities were identified. We initially excluded insulin-dependent hypoglycemia as well as hypokalemia resulting from urinary potassium loss or aldosterone effect. Following a hospital-wide multidisciplinary consultation and thorough assessment of the patient’s condition, we hypothesized that the patient was likely experiencing a rare paraneoplastic syndrome, specifically Doege–Potter syndrome. We determined that preoperative tumor vascular embolization followed by surgical resection represented the optimal treatment strategy.

**Figure 4:**
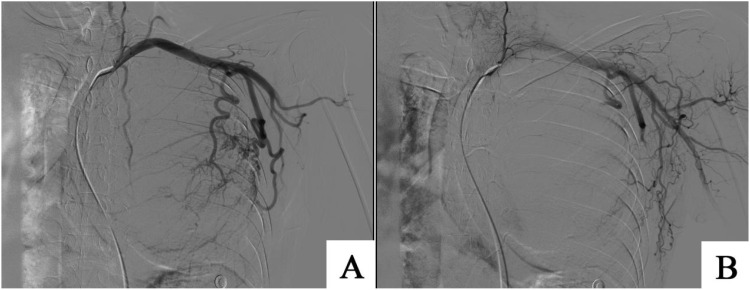
**(A)** Using the improved Seldinger technique to puncture the right femoral artery, a 5F catheter was used to select the left thoracic external artery, with the blood supply artery being the left thoracic external artery. Distal disrupted tumor blood vessels were visible in the internal mammary artery. **(B)** Using 1000 μ m gelatin sponge to embolize the main blood supply vessels of the tumor, reconstruction showed that most of the distal blood vessels of the tumor were occluded, and the blood supply was significantly reduced.

**Figure 5:**
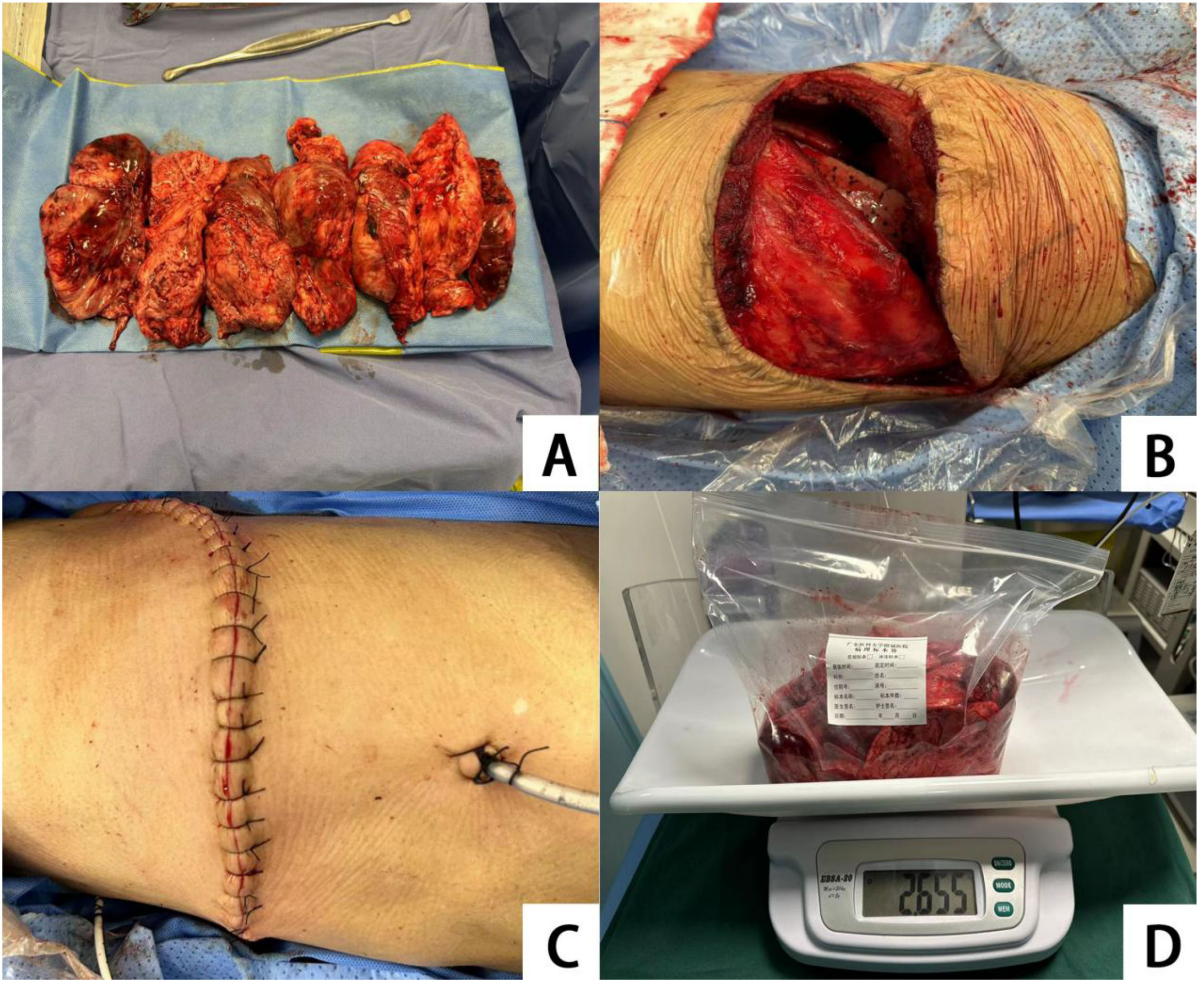
**(A)** Intraoperative spindle shaped block resection of the tumor is shown; **(B)** The left lung shows good recovery after tumor resection; **(C)** The suture situation of the incision after closing the chest layer by layer is shown; **(D)** The weight of the tumor removed during the surgery is 2.655 Kg.

On September 14, 2024, with assistance from the Interventional Radiology Department, embolization of the tumor’s primary feeding vessels was performed (**Figure 4**). On September 18, 2024, under general anesthesia and with support from the Anesthesiology Department, the patient underwent surgical resection of a solitary fibrous tumor of the left chest wall, combined with a left upper lobe wedge resection and partial resection of the left fifth rib. A posterolateral incision along the fifth rib was made, and the chest was entered via the rib bed. The tumor was resected en bloc in a fusiform manner, and the tumor pedicle, located at the apex of the left parietal pleura, was excised (**Figure 5**). The procedure was uneventful, with an estimated intraoperative blood loss of approximately 200 mL. Postoperatively, the patient was safely transferred to the ward. Lung re-expansion was satisfactory, and follow-up measurements of blood glucose and serum potassium levels returned to normal. As of the last follow-up on September 21, 2025, the patient exhibited excellent postoperative recovery, with no significant abnormalities noted during outpatient evaluations. The patient will undergo indicated imaging surveillance for long-term follow-up.

## Comment

Solitary fibrous tumor (SFT) is a rare mesenchymal neoplasm, predominantly benign in nature. However, its rare paraneoplastic manifestation, Doege-Potter syndrome (DPS), is characterized by non-insulin-dependent refractory hypoglycemia (9). Studies indicate that the hypoglycemic mechanism in DPS is closely associated with the secretion of high-molecular-weight insulin-like growth factor-II (IGF-II) by the tumor. This molecule preferentially binds to IGF-binding protein-2 (IGFBP-2), forming an abnormal binary complex that interacts with insulin receptors, thereby inducing hypoglycemia. The insulin-like activity of IGF-II enhances glucose transport while inhibiting hepatic gluconeogenesis and lipolysis, further exacerbating hypoglycemia. Additionally, IGF-II can induce hypokalemia by promoting the intracellular shift of serum potassium (5). These findings were corroborated in our case, where routine glucose infusion and potassium supplementation failed to stabilize the patient’s blood glucose and potassium levels. Notably, surgical resection of the tumor reversed both hypoglycemia and hypokalemia. Although our institution does not routinely assay serum IGF-II or the IGF-II/IGF-I ratio, the patient presented with refractory fasting hypoglycemia that required continuous glucose infusion and was not accompanied by any clinical or biochemical evidence of hyperinsulinemia. Moreover, both hypoglycemia and hypokalemia resolved immediately after complete tumor resection; this “surgical cure” phenomenon is characteristic of IGF-II-mediated hypoglycemia and concomitant hypokalemia. Consequently, despite the absence of direct biochemical confirmation, we consider the diagnosis of Doege–Potter syndrome and the proposed IGF-II-driven pathophysiology to be both reasonable and consistent with the published literature.

Multidisciplinary team (MDT) consultation provided comprehensive support for the management of this case. Existing literature highlights the advantages of MDT in the diagnosis and treatment of complex diseases (10–12). In this patient, the large tumor size precluded immediate surgical resection. Previous studies have suggested that anti-angiogenic targeted therapies may inhibit SFT growth (13). In this patient, pathology revealed nuclear STAT6 (+) and high CD34 expression. STAT6 translocates to the nucleus and directly binds to the promoters of VEGFA, VEGFC and PDGFB, establishing a self-sustaining transcriptional amplification loop that results in dense microvasculature and hypoxia-driven further angiogenesis (14, 15). SFTs are reported to over-express VEGFR-2/3 and PDGFR-β (16–18); to halt tumor progression, we combined an anti-angiogenic targeted agent with chemotherapy. This strategy suppresses neovascularization, reduces hypoxia-induced IGF-II secretion, enhances CD8^+^ T-cell infiltration, and creates a dual vascular-immune inhibition of the tumor microenvironment, thereby reversing tumor growth. Anlotinib, a multi-target anti-angiogenic agent approved in China for soft-tissue sarcoma, offers robust efficacy and a well-characterized safety profile, and it acts synergistically when combined with chemotherapy in this setting (19, 20). Consequently, we attempted three cycles of anlotinib combined with paclitaxel and cisplatin. However, the therapeutic benefit was limited, underscoring the need for further clinical research to optimize systemic treatment options for chest wall SFT.

Hemorrhage is a major risk during surgical resection of large chest wall fibrous tumors. Preoperative interventional embolization of tumor vessels significantly reduces intraoperative blood loss, ensuring a clear surgical field and enhancing procedural safety (21–23). However, post-embolization tumors may reestablish blood supply through collateral circulation. The timing of collateral revascularization varies depending on tumor type, location, and individual patient factors, with no standardized timeframe currently established. Therefore, surgical resection should be performed promptly following embolization to minimize the impact of collateral circulation. In this case, preoperative embolization not only effectively controlled hypoglycemia and hypokalemia but also optimized conditions for subsequent surgical intervention.

In conclusion, chest wall SFT associated with Doege-Potter syndrome represents a rare and complex clinical entity. Surgical resection remains the cornerstone of treatment, while MDT collaboration facilitates the development of tailored diagnostic and therapeutic strategies to address challenges such as large tumor size and intraoperative risks. Future research should focus on elucidating the pathogenesis of SFT and refining treatment approaches to provide robust evidence for clinical practice.

## Data Availability

The raw data supporting the conclusions of this article will be made available by the authors, without undue reservation.
